# The Role of Central Complex Neurons in Prey Detection and Tracking in the Freely Moving Praying Mantis (*Tenodera sinensis*)

**DOI:** 10.3389/fncir.2022.893004

**Published:** 2022-06-13

**Authors:** Anne Wosnitza, Joshua P. Martin, Alan J. Pollack, Gavin J. Svenson, Roy E. Ritzmann

**Affiliations:** ^1^Department of Biology, College of Arts and Sciences, Case Western Reserve University, Cleveland, OH, United States; ^2^Department of Biology, Colby College, Waterville, ME, United States; ^3^Cleveland Museum of Natural History, Cleveland, OH, United States

**Keywords:** predator, central complex, target detection, movement control, praying mantis, extracellular recording

## Abstract

Complex tasks like hunting moving prey in an unpredictable environment require high levels of motor and sensory integration. An animal needs to detect and track suitable prey objects, measure their distance and orientation relative to its own position, and finally produce the correct motor output to approach and capture the prey. In the insect brain, the central complex (CX) is one target area where integration is likely to take place. In this study, we performed extracellular multi-unit recordings on the CX of freely hunting praying mantises (*Tenodera sinensis*). Initially, we recorded the neural activity of freely moving mantises as they hunted live prey. The recordings showed activity in cells that either reflected the mantis's own movements or the actions of a prey individual, which the mantises focused on. In the latter case, the activity increased as the prey moved and decreased when it stopped. Interestingly, cells ignored the movement of the other prey than the one to which the mantis attended. To obtain quantitative data, we generated simulated prey targets presented on an LCD screen positioned below the clear floor of the arena. The simulated target oscillated back and forth at various angles and distances. We identified populations of cells whose activity patterns were strongly linked to the appearance, movement, and relative position of the virtual prey. We refer to these as sensory responses. We also found cells whose activity preceded orientation movement toward the prey. We call these motor responses. Some cells showed both sensory and motor properties. Stimulation through tetrodes in some of the preparations could also generate similar movements. These results suggest the crucial importance of the CX to prey-capture behavior in predatory insects like the praying mantis and, hence, further emphasize its role in behaviorally and ecologically relevant contexts.

## Introduction

Complex movements, even in supposedly simple animals like insects, are influenced by various brain circuits. This can include sensory-guided movements such as a locust walking on discontinuous substrates (Niven et al., 2010), learned behaviors (Ofstad et al., 2011), navigational behaviors such as path integration (Green et al., 2017; Stone et al., 2017; Turner-Evans et al., 2017), and foraging movements in an arena (Martin et al., 2015). The central complex (CX) is a brain region that has received attention for its role in controlling complex behaviors (Pfeiffer and Homberg, 2014). This highly conserved set of midline neuropils includes the protocerebral bridge (PB), fan-shaped body (FB) [also called the upper central body (CBU)], ellipsoid body (EB) [also called the lower central body (CBL)], and two paired noduli (Supplementary Figure S1). The PB, FB, and EB are unique for being divided into distinct columns or wedge structures that have been found to include spatial information used in navigation. Considerable information is now available about sensory inputs to these neuropils. Much of the data have focused on detection of polarized light (Heinze et al., 2009). Polarized light-sensitive cells in locusts project to the PB in a regular manner that describes a map of polarized sky light (Heinze and Homberg, 2007). Polarized light mapping has also been reported in numerous other insects including dung beetles (el Jundi et al., 2015), monarch butterflies (Heinze and Reppert, 2011), and *Drosophila* (Warren et al., 2019). In addition, non-polarized directional light responses were found in locusts (Rosner and Homberg, 2013; Pegel et al., 2018), cockroaches (Kathman et al., 2014), *Drosophila* (Seelig and Jayaraman, 2013), and monarch butterflies (Heinze and Reppert, 2011). Mechanical inputs from the antennae have been documented in cockroaches (Ritzmann et al., 2008) and flies (Currier et al., 2020; Okubo et al., 2020) as well as from halteres of flies (Kathman and Fox, 2019).

The directional properties in sensory studies have led to consideration of navigational control that has uncovered head direction cells in *Drosophila* (Seelig and Jayaraman, 2015) and cockroach (Varga and Ritzmann, 2016). In addition, the existence of ring attractor pathways between the PB and EB have been demonstrated in *Drosophila* that can integrate angular motion and external visual compass cues into a coherent head direction signal. This signal can be used as one of the inputs to a path integrator (Green et al., 2017; Turner-Evans et al., 2017). Indeed, a path integration model has been developed based on CX recordings and structures in bees (Stone et al., 2017).

Any kind of navigational control requires that the CX affects motor systems, and motor effects have been demonstrated in the CX. Genetic manipulations that resulted in damage to the PB resulted in walking deficits (Strauss, 2002). Extracellular recordings from tethered cockroaches revealed CX neurons that control step frequency (Bender et al., 2010) and turning movements toward a target (Guo and Ritzmann, 2013). In freely walking cockroaches, such recordings revealed several neurons that are active just before turning or forward acceleration (Martin et al., 2015). These data contributed to 2 dimensional maps of forward movement or turning associated with each neuron. The overlap among 2D representations of CX cells suggests a population code that can influence movement in any direction. This control occurs at least in part by altering reflexes to specific joints in the thoracic ganglia (Martin et al., 2015).

Navigational studies suggest that the CX serves to control directional motion. Indeed, a recent study indicates that it plays a role in goal-directed movements (Green et al., 2019). As important as this study was in revealing moment-to-moment neural adjustments, it implied that the fly was moving toward a goal by the fact that it maintained a specific angular bearing as it walked (menotaxis). This is in contrast to recordings in bats that revealed goal-directed cells in the hippocampus that signal the bat's heading toward the location of a specific goal (Sarel et al., 2017).

Predatory insects such as praying mantises (Prete, 1999) and dragonflies (Olberg et al., 2005; Mischiati et al., 2015) provide systems that are similar but different from the above-described navigational behaviors. Rather than foraging movements followed by return to a nest, predators must target a specific easily-identified goal and move toward that goal before making a very precise strike. Failure to precisely guide movements toward the prey means that predators will not eat. Praying mantises employ a unique 3-dimensional visual system to establish distance parameters (Nityananda et al., 2018). Neural correlates for this stereopsis have been identified in the mantis brain (Rosner et al., 2019). For various mantis species, a range of visual stimulus parameters including, among others, size, background contrast, leading edge length, speed, location in the visual field, and relative direction of movement evokes predatory responses (Prete et al., 2011). Regardless of cues, the praying mantis uses this information to stalk and eventually strike the prey. The actual hunting strategy varies among species from active stalking (Prete et al., 2012) to ambush (Inoue and Matsura, 1983). In the so-called “generalist” species, the hunting strategy can switch from stalking to ambush as the praying mantis feeds (Inoue and Matsura, 1983; Bertsch et al., 2019), and the switch can be mimicked by injection of insulin (Bertsch et al., 2019).

Various components of the praying mantis hunting strategy imply considerable high-level control, much of which is reminiscent of navigational control properties seen in other insects. The visual guidance necessary for tracking should rely on neurons that are tuned to specific azimuth angles around the insect's head. Such neurons have been shown to be present in the CX of locusts (Pegel et al., 2018), monarch butterflies (Heinze and Reppert, 2011), and dung beetles (el Jundi et al., 2015). Directed movements are reminiscent of cockroach motor control (Guo and Ritzmann, 2013; Martin et al., 2015) although much more precise. Even the hormonal control of changes in hunting strategy by satiety and insulin suggests modulatory properties such as those seen in the CX of locusts (Nässel and Homberg, 2006) and *Drosophila* (Kahsai et al., 2010). If the sensorimotor control of praying mantis hunting can be shown to be controlled in the CX, it would be possible to directly examine the neural control of both the precise sensory and motor aspects of this behavior in CX neuropils as well as the necessary sensorimotor transformations and context dependent modifications that might occur there.

To examine the role of the CX in predation, we recorded extracellularly from the CX of freely moving praying mantises (*Tenodera sinensis*) as they stalked prey. We began with live prey to establish that CX activity is associated with real predatory behaviors. We then developed a simulated prey system that allowed us to generate reproducible targets around the mantis for quantitative analysis of both sensory and motor aspects of the behavior. In both situations, our recordings revealed units that fired in response to prey or moving targets presented at specific angles and distances around the subject, as well as neurons that became active just before turning or forward motion. These data suggest that neurons in the CX could control the hunting and targeting movements of the mantis. Some neurons appeared to be involved in both sensory and motor aspects of the targeting behavior. Thus, the CX does appear to play a role in controlling the predatory behaviors in this species of praying mantis.

## Methods

Adult praying mantises (*Tenodera sinensis*) from a laboratory colony were used in all the experiments. The mantises were housed in individual plastic containers and given food and water *ad libitum*. They were kept in a 12/12-h light/dark cycle at 27°C. After the final molt, only 14- to 17-day-old healthy females were chosen for the experiments.

### Animal Preparations and Recording

The insects were first anesthetized with ice. After they stopped moving, they were restrained ventral side down against a flat silicone surface with insect pins bent into a hook that surrounded them but did not penetrate any part of them. A plastic collar was positioned around the neck to support the head, and dental wax was placed around the head to stabilize it. The preparation was transferred into a plastic container, and ice was placed around the animals to minimize hemolymph flow and body movements, which could interfere with wire implantation. A small window between the antennae was cut into the cuticle and removed over the brain. Connective tissues and fats were carefully removed to expose the brain. The sheath surrounding the brain was opened mechanically in a small area dorsal to the central complex, and a small amount of saline (Blagburn and Sattelle, 1987) was placed in the head capsule to cover the brain tissue.

One or two wire-bundle tetrodes were used for recording. Each tetrode consisted of four 12-μm nichrome wires (Kanthal RO-800; Sandvik Heating Technology, Hallstahammar, Sweden) twisted together. The tetrode wires were connected to an adaptor and secured in a Delrin and epoxy package. Before each experiment, the tip of each tetrode was cut, polished, and plated with copper such that it had a regular arrow shape and starting impedance of between 0.5 and 1.5 MΩ.

With the brain exposed, the tetrode was inserted into the brain with a micromanipulator, and the adaptor was mounted in the headstage of a Neuralynx Cheetah (Bozeman, MT, United States) digital interface. A separate larger-diameter (56 um) insulated copper wire was inserted into an anterior location in the pronotum to serve as a reference/ground electrode. The tetrode was fixed in the brain at the location that had the best signal:noise ratio and where the units responded to sensory stimuli (i.e., light on or off and/or antennal contact). The head capsule was then covered and sealed with blue-light curable clear glue (Loctite 3,555 transparent Light Cure Adhesive) to anchor the tetrode wires in place, taking care to not obscure the compound eyes and ocelli. A plastic tether was glued to the posterior pronotum and the tetrode (s), and the reference electrode was secured along the pronotum and the tether with the same glue as used on the head as well as with dental wax. Next, constraints were carefully removed, and the animals were transferred into a clear acrylic arena positioned on top of an LCD screen. The animals were given at least 60 min to recover from the cold anesthesia. All the experiments lasted between 2 and 4 h depending on the quality of the neural recordings and preparation.

Videos were either captured at 30 or 120 frames s^−1^ with a Casio Exilim HS camera or at 40 or 100 frames s^−1^ with a Point Gray video camera. The cameras were positioned centrally above the arena so that the entire arena was visible and all movements could be seen. The position of the live or simulated prey and the mantises' head and body position and orientation in the arena were tracked using the DLT tracking software from MATLAB (Hedrick, 2008). From the position and orientation of the mantis' head, the visual field could be defined, and the angular position, size, and velocity of the prey in the field were measured. This allowed us to correlate the stimulus movement to the resulting electro-physiological and locomotor responses.

The data from the Neuralynx system were saved directly to a PC. For each electrode, the collected data included voltage waveforms and time stamps that marked the point in time where an action potential that exceeded a pre-set threshold occurred within a given data file. The data also included synchronization pulses to link the Neuralynx time with coincident high-speed digital video recordings.

### Live Prey Experiments

After the tetrodes were placed in the their brain, the subjects were placed in an arena and allowed to recover. Up to 4 cockroach nymphs were then placed in the arena, and both the mantises and the prey were allowed to move freely while their activity was recorded from the tetrodes. Movements were recorded with the camera situated above the arena. Frame-by-frame movements of both the prey and the mantises were quantified offline. After the spikes recorded from the tetrode had been sorted (see below), individual unit time stamps could then be associated with either the mantises' movement or a prey individual that a mantis was targeting.

### Simulated Prey System

In order to obtain quantitative data relating brain activity to movement, we developed a simulated prey system that could generate targets at reproducible distances and angles around the mantis' head. Using a computer screen under the transparent arena floor, we could exploit previous observations that praying mantises would target and strike at moving images on a computer screen (Prete et al., 2013). We reasoned that placing a computer screen under the floor of the arena would allow us to repeatedly present the subject with simulated prey at predetermined distances and angles. A custom MATLAB routine generated the simulated prey at various angles around the subject at preset distances. The circle in which the simulated prey appeared could be moved on the screen so that the praying mantis was always kept in the center of the presentations. The simulated prey consisted of a 2 × 1 cm black ellipse that moved back and forth in parts of the circle with the mantis in the center. The target moved at 2 cm/s (Supplementary Video S1).

Again, CX activity was recorded from 1 or 2 tetrode bundles that were inserted into the brain targeting the CX. In all, we recorded 99 units from 10 preparations where the tetrode location could be determined histologically (Supplementary Figure S1). Eight of the preparations used a single tetrode implant. The remaining 2 preparations had 2 tetrodes inserted for a total of 12 tetrode locations. Eight of these tetrodes (68 units) were located in the FB of the CX, while 4 (31 units) were located in the mushroom body (MB). Two of the MB placements were in the peduncle and two in the medial lobe. Because there were few MB recordings, we did not analyze them further but present the data and some contour plots (Supplementary Figure S3) for completeness.

The high-speed video camera placed above the arena monitored the praying mantis's movements relative to the simulated prey. When the praying mantis attended to the prey, it turned its head toward the target. This action could include neck movements, rotation of the T1-T2 thoracic joint, or very large turns including leg movements that rotated the body toward the prey. We digitized the movements of the praying mantis and the simulated prey and combined the records with the sorted activity of single CX units. In some preparations, we also injected currents through the tetrodes at the end of the experiment and monitored resulting mantis movements.

The praying mantises readily targeted, stalked, and struck the simulated prey appearing 2.5, 5, 7.5, 10, and 12.5 cm from them. Beyond 12.5 cm, the attention dropped off. We suggest that this was because the flat screen image on the floor was no longer seen by the subject. In support of this, another study using the same arena showed no similar drop off to presentation of a dead cockroach nymph that provided a 3-dimensional image.

### Spike-Sorting Analysis

A single unit analysis was performed off-line in Spike2 v7.15 (CED, Cambridge United Kingdom). To sort multi-unit activities into single unit activities, we conducted user-supervised semi-automated tetrode template matching and K-means assisted principal component analysis. Clusters with > 3% of all spike events falling within the 2-ms inter-spike-interval criterion were excluded from analysis, because this suggested that we were not monitoring a single unit. Only single-unit activity with stable amplitude was used for later analyses. Time stamps of all the units were exported and loaded into custom MATLAB scripts for further analysis.

### Analysis of Spike Activity Relative to Target and Mantis Movements

**Figure 1:**
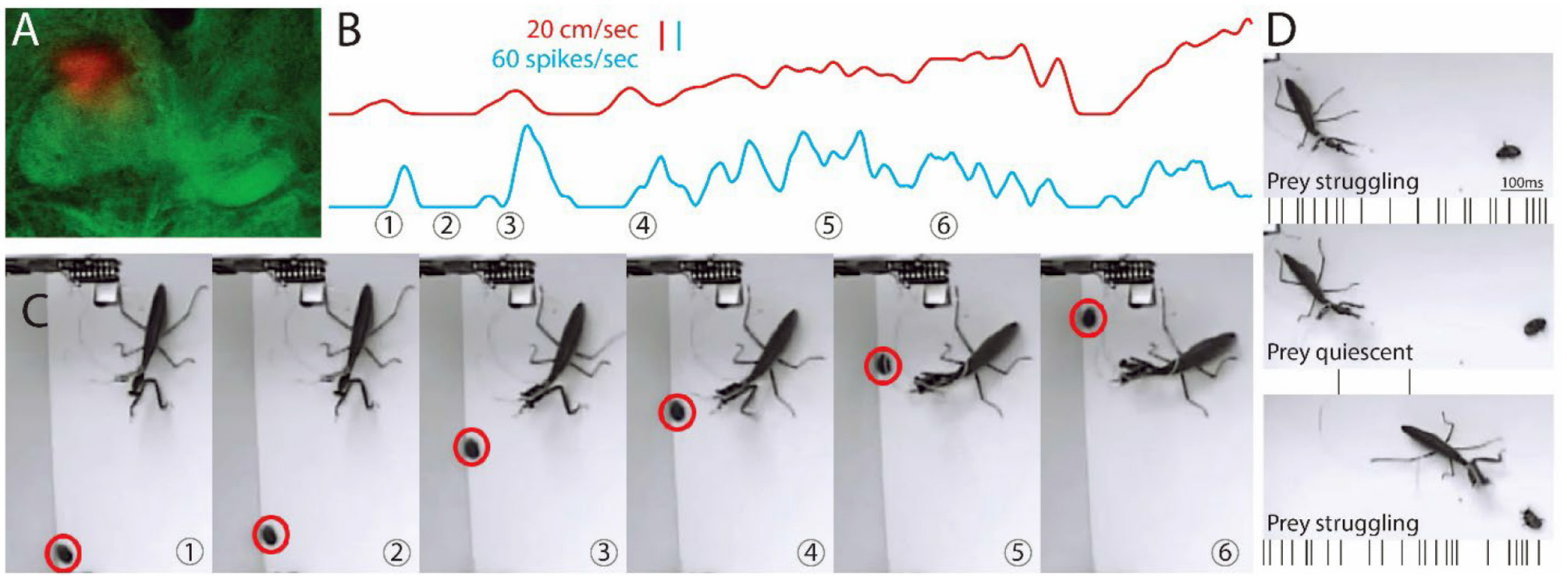
Images from video and recording of a single unit during hunting behavior. **(A)** Location of a tetrode is shown in the dorsal region of the FB on the mantis's right side. **(B)** Shows the activity of the cell (blue) along with the movement of the prey (red). **(C)** Sample frames from Supplementary Video S1 are shown with time of frame numbers indicated in **(B)**. **(B,C)** Prey starts to move in first frame. Central complex (CX) unit activity increases and then stops when the prey stops (2nd frame). In frame 3, the prey runs as the mantis tracks it, and CX unit activity increases and is maintained through frame 6. Before the mantis tracks the prey, the single CX unit is correlated with mantis movement (r > 0.7, sliding Pearson's coefficient). After the mantis orients toward the prey, the same unit is correlated (r > 0.7) with prey movement. **(D)** Another prey is on its back and struggles (top) and then stops (middle) and struggles again just before the strike. Activity, shown here as spikes below each frame, is parallel to movement.

Once we obtained the time stamps for each unit, they could then be analyzed relative to target movement or that of the mantis toward the target. In the case of live prey trials (e.g., Figure 1), this involved comparing the unit's spike frequency to the prey's movement velocity. In trials involving simulated targets, a much more involved analysis (described below) related spike activity to target movement and/or to the mantis's own movement. In either case, the simulated trial analysis relied on two steps. First, raster plots were constructed relative to the action that was being examined (target or mantis movement). Then, in order to achieve statistical significance in the relationship between spike activity and either target or mantis movement, the data were subjected to spatial temporal receptive field (STRF) analysis of movement.

Analysis of the responses of each identified unit (neuron) proceeded from the aligned time stamp vectors of spikes in each cell and the per-frame location in Cartesian coordinates of the mantis's head and prothorax, and the prey. The angle between the head and the prothorax (“head angle”) was calculated from the angle between a line defined by points on the outer edge of each eye and another line defined by points on the prothorax at the neck joint and the joint of the mesothorax.

To describe the prey stimulus, we first translated and rotated the cartesian coordinates of the head and a point at the center of the prey target to center the head in the new coordinate frame and align the center of the visual field along the positive y-axis. This allowed us to continually represent the location of the prey relative to the visual field of the mantis in every frame of the video, as the mantis moved and turned its head during the trial. In this new coordinate frame, we calculate the Euclidean distance between the prey and the mantis's head (“prey distance”) and the angular location of the prey in the visual field (“prey angle”).

We produced raster plots and peri-stimulus time histograms (PSTHs) describing the neuron's response to two stimuli: the prey angle and the head angle. For the head angle raster plots, we used a threshold of 2 standard deviations (SDs) above the mean head angle velocity to identify the time when saccades were initiated. For the prey angle plots, we selected a particular visual angle for each cell (see below and in Results for details on how this angle was chosen). The rasters and PSTHs were triggered at the time a head saccade began or when the prey crossed the chosen angle. We calculated the mean of the instantaneous firing rate over all events for each stimulus type using a bootstrapping procedure to calculate a 95% confidence interval (2 SDs). To determine whether a cell responds to the stimulus, we used a threshold procedure. We randomly selected segments of the spike time vector of the same length as the event window (1 s) and the same number of segments as recorded events. We calculated the mean and SD of the firing rate across the segments and defined a threshold of the mean ± 2 SDs.

To fully characterize the responses of the cells to both the animal's own movement and the visual stimulus of the prey, we used a reverse correlation technique designed for natural stimuli (Theunissen et al., 2001) (see also http://www.strflab.berkeley.edu/), briefly summarized here and in detail below. This method estimates the correlation between a stimulus and the spiking activity of a neuron using a generalized linear model. The model is statistically verified by minimizing the mean squared error between the predicted response to a test portion of the recording and the recorded response. Because we are extending this method to a freely moving animal, the stimulus and response of the animal are necessarily non-random and autocorrelated, increasing the possibility of false-positive correlations. We, therefore, additionally validated the statistical significance of a neuron's response using a shuffle procedure, offsetting the stimulus and response in time, to generate the expected value and standard deviation of correlations attributable to chance.

The reverse correlation method estimates the spatiotemporal receptive field (STRF) for linear-nonlinear models of neurons (Supplementary Figure S2). Spike times were binned for each video frame to match the sampling rate of the stimuli. The prey position, in head-centered coordinates, was binned into a 31 × 31 matrix for each frame. The head velocity was binned into a 21 × 1 vector covering the range of observed head velocities from right (negative bins) to left (positive bins) saccades. For each frame, the bins for the current prey location (x,y; cm) and head velocity (rad/s) are occupied by a 1 and the remainder by zeros.

The response of each neuron to this combined prey and head movement stimulus matrix was estimated using a generalized linear model. The model incorporates a linear transformation of the stimulus by an STRF, followed by an exponential (Poisson) nonlinearity [modified from Talebi and Baker (2012)]:


$$\begin{array}{lll} w\left(t\right) & = & \;\overset{M}{\underset{i=1}{\sum }}\overset{N}{\underset{j=1}{\sum }}\overset{O}{\underset{k=1}{\sum }}h\left(i,j,k\right)s\left(i,j,k-\right)\\ r\left(t\right) & = & \;e^{w} \end{array}$$

where s(i,j,k) = the stimulus matrix (size M,N) for the i,j^th^ “pixel” at the k^th^ delay (in frames); h(i,j,k) is the corresponding linear filter (STRF) weight; w(t) = response of the linear filter as a function of time (t); r(t) = estimated model response as a function of time (t). We conducted scaled conjugate gradient optimization, implemented in the strflab interface, to optimize the STRF weights h(i,j,k) and minimize the mean squared error between the estimated model response r(t) and the response measured from the neuron. Delays were chosen to cover time lags from −0.5 to 0.5 s.

The STRF was separated into a prey location matrix of 31 × 31 × 16 and a head velocity matrix of 31 × 16. The weight values can be analogized to positive or negative correlation between either the prey location or the head velocity stimulus and a spike at the 0-time delay. To establish a threshold for significant correlation, we used a shuffle procedure. The stimulus and spiking response vectors were each shifted by a random amount, removing the temporal correlation between them but preserving the temporal characteristics of each. STRFs were fitted to the shuffled data. This process was repeated 10 times, and the mean and SD of the STRF weights was calculated. We used a significance threshold of mean weight ±2 SD for each bin of the STRF matrix. These were presented as contour plots surrounding contiguous bins that exceeded the threshold for each time delay.

Because prey movement and head saccades to center the prey in the visual field are naturally correlated, we performed additional analyses to characterize the responses of the cells. We filtered out periods of the recording where head movement and prey movement overlapped by 0.5 s to isolate data where the head or the prey were moving alone. We then produced STRFs for the “head alone” and “prey alone” data subsets. Only cells with STRFs that still exceeded the threshold in one or both of the alone conditions were identified as head-movement selective or prey-movement selective.

Finally, for each cell with STRF weights that exceeded the threshold, we identified the maximum weight across all delays. We plotted the time series of the weights at this maximum prey position or head velocity across all delays. The same thresholding procedure was used to identify periods of significant correlation in the time series. Points to the left of zero represent movement that occurs preceding spikes, and points to the right represent movement that follows spiking. This means that unlike the raster displays that showed spike activity relative to target or mantis movement at the 0 point, in STRF plots, change in spike activity was taken as the zero point, and movement was plotted before and after that point. Therefore, for example, if target movement was being analyzed, a positive peak to the left of zero would indicate that the unit showed an increase in activity after the target moved. A negative peak would suggest that the unit activity decreased after the target moved. Either of these results would be consistent with a sensory response to the target movement. In contrast, a significant peak to the right of the zero point would suggest that the increase in spike activity in that unit preceded the movement. This occurred when analyzing activity changes relative to the mantis's own movement (such as toward the target) and suggested that the cells could be involved in generating such movements.

**Figure 2:**
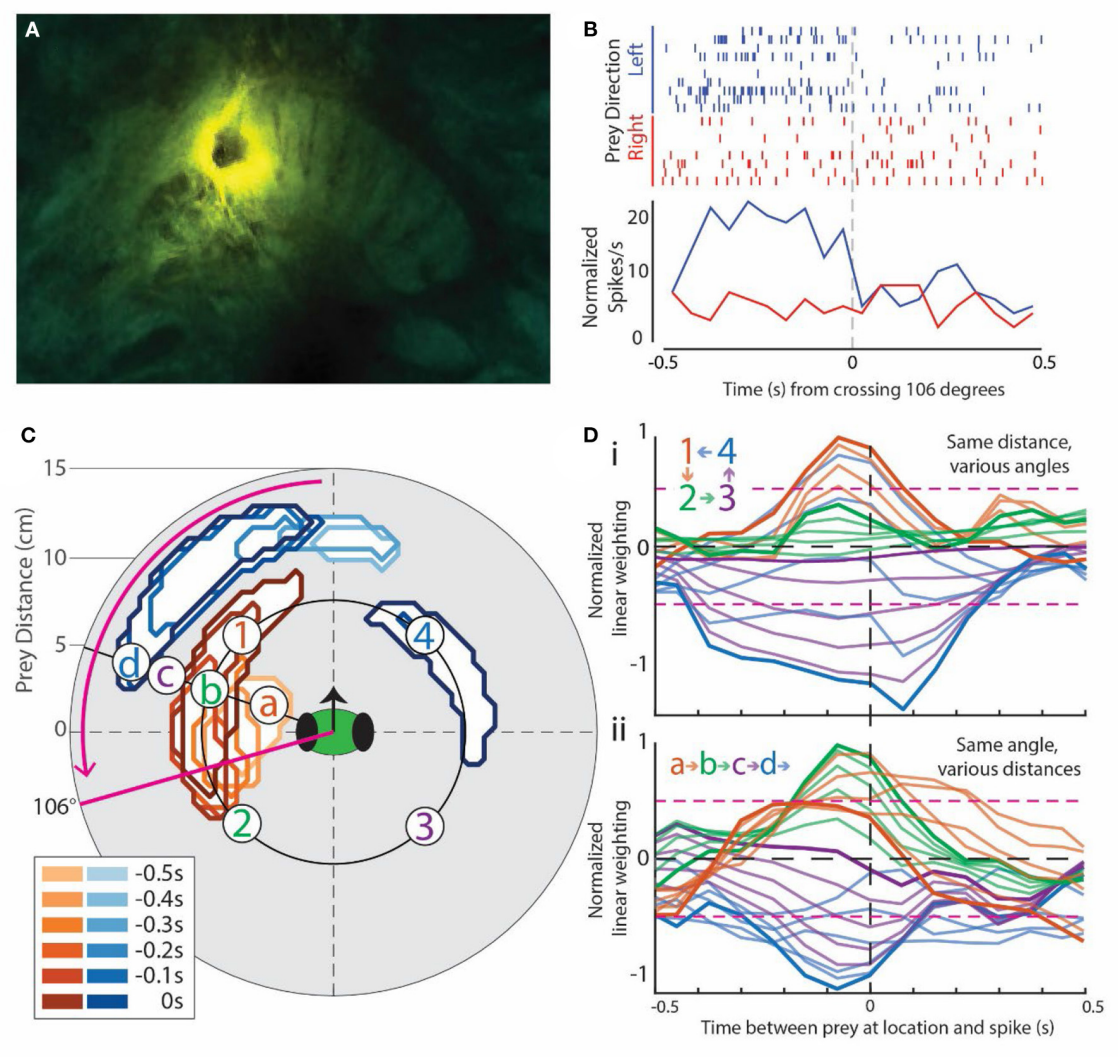
Example of prey-motion-responsive cell. All data in this Figure are from the same cell located in the left fan-shaped body (FB). It was chosen because it exemplified the most common subtype of prey-responsive cells and direction selectivity, and it had clear positive and negative correlation regions in the visual field. **(A)** Image of the central complex showing the lesion dye from the electrode on the mantis's right-side FB. **(B)** Rasters (top) and peri-stimulus time histogram (PSTH, bottom) for the angle at which prey movement elicited the greatest response in this cell, which, in this case, was back and forth around 106° to the left of the mantis's forward-facing visual field [refer to the solid magenta line in **(C)**]. Trials are split into the left- (blue) and right-moving prey (red). The corresponding PSTHs were calculated as the sum * (spikes in bin for all trials)/(number of trials) * (length of the bin). This normalized each PSTH for unequal numbers of left and right trials (see Methods for more detailed explanation). **(C)** Contours outlining the positions of the moving prey with significant weights in the generalized linear model for this cell. Warm colors indicate regions with positive weights (analogous to positive correlation), and cool colors indicate negative weights. The inset box is a legend indicating the delay between prey in the area outlined by the contour (lighter colors for longer delays) and a spike in this cell. In effect, a prey stimulus in the outlined area is associated with a spike at some time delay later. The magenta arrow indicates the direction of prey movement that produced a larger response in the PSTH in **(B)**. **(D)** Spatial temporal receptive field (STRF) plots taken from various locations in the contour plot in **(C)**. A peak to the left of 0 that rises above the 2-SD level (dotted magenta horizontal lines) indicates a strong correlation with movement before an increase in spike activity. A peak that goes below the lower 2 SD line indicates a negative correlation between movement and activity (decrease in spiking). **(D**i**)** includes samples from 4 numbered regions indicated in **(C)** around the same distance. The color of each curve matches that of the site indicated in **(C)** (labeled a–d). Darker curves are the correlations for prey at the indicated points, and the lighter curves are the correlations at equally spaced points between these points. Light brown lines are at points taken between the points labeled 1 and 2, light green lines from points between 2 and 3, and so on. This progression is indicated in the insert on the plots. **(D**ii**)** shows lettered responses to sites at the same angle relative to the head of the mantis but at different distances. In this case, sites at which the curves are recorded are indicated as letters on the contour map in **(C)** and colors of the STRF curves match those of the letters in **(C)**.

We examined the STRF plots relative to presentations at numerous angles and distances around the mantis and then displayed them as contour plots relative to the mantis's head (e.g., Figures 2C,D). Positive increases were plotted as brown regions and negative changes as blue regions. Delays in STRF plot peaks relative to the zero point were coded as variations in blue or brown color, as indicated in a legend for the plot.

### Histology

Prior to insertion, the tip of the tetrodes was dipped into DiI (DiCarlo et al., 1996), so that its location in the brain could be established histologically after the experiment. Additionally, at the end of each experiment, a 5-mA, 5-ms DC current was applied between the tetrode wires and the reference electrode in order to deposit copper at the recording sites. The brains were then removed and placed in a 20% ammonium sulfide/saline solution for 15 min to precipitate the copper and then rinsed twice in 0.1 M phosphate-buffered saline (PBS). After fixation with 4% paraformaldehyde and 0.25% glutaraldehyde, the brains were again rinsed twice in PBS and dehydrated in an increasing ethanol series (50, 70, 90, 95, and 2 × 100%, 20 min each). The brains were then transferred to a mixture of methyl salicylate and ethanol (1:1), followed by 100% methyl salicylate (45–60 min each). Finally, the brains were mounted in a DPX mounting medium (Electron Microscopy Sciences) between two glass cover slides separated by spacing rings to avoid compression. Whole mounts were scanned with a confocal laser microscope (Leica TCS SP8 gated STED) equipped with a 10 × objective (HC PL APO 10 × /0.4 dry CS; Leica, Bensheim, Germany) to determine the location of DiI 488 in the brain. Confocal stacks were analyzed offline with the ImageJ software (Schindelin et al., 2012).

In cases where the fluorescent whole mount preparation gave inconclusive results, the copper method was used. The mounting medium was removed using xylene. The brains were then embedded in Paraplast and sectioned at 12 um. The sections were run by Timm's sulfide-silver intensification (Tyrer and Bell, 1974) and then fixed, dehydrated, and covered for imaging. Concentrated brownish deposits occurring in several adjacent serial sections were identified as tetrode locations. Copper deposits coupled with DiI provided strong localization of tetrode locations.

## Results

### CX Unit Activity With Live Prey

We implanted a set of tetrode wires into the brain of 4 individual praying mantises, targeting the CX and then, after a period of recovery, released the subjects into an arena with 4 cockroach nymphs that provided natural targets. CX activity was sorted into individual units, and their activity was examined relative to prey movement. In the example shown in Figure 1, the tetrode was located in the dorsal right FB (Figure 1A). Initially, the activity in the unit shown here increased in association with the praying mantis's turns or forward movements (Supplementary Video S2). This pattern was consistent with what had been seen previously for cockroach (Martin et al., 2015). The praying mantis eventually began to attend to one of the prey. When this occurred, the same unit's activity increased now in conjunction with prey movement (Figures 1B,C and Supplementary Video S1). Later, the praying mantis attended to a cockroach nymph that was on its back and struggling to right itself. As long as the prey struggled, the CX neuron was active (Figure 1D). However, when the nymph ceased movement, the CX neuron became silent and only returned to activity when the nymph began struggling and continued as the praying mantis stalked and ultimately struck the nymph. Interestingly, as the stalking took place, another nymph walked through the praying mantis's field of view and was ignored by the CX unit that was reporting on the first prey's movements, suggesting a form of selective attention. The recordings for this preparation yielded 8 units, and 3 of these had similar responses to prey movement. The other three preparations yielded 2 out of 5, 1 of 4, and 2 of 6 prey responsive units.

### Selective Responses to Simulated Prey Position

In order to obtain quantitative data on CX activity relative to mantis stalking behavior, we switched from live prey to the simulated prey system. As indicated in Methods, these experiments were performed on 10 different praying mantises using a total of 12 tetrode implants. The involvement of CX units in prey stalking requires three properties. First, CX units must be seen to track prey at various distances and angles around the mantis. Second, CX units must show indications that they control movement, as has been demonstrated in cockroach (Martin et al., 2015). Third, at least some CX units should be seen to be associated with actual orientation movements toward a tracked target. We will begin our analysis with activity associated with tracking prey.

Figure 2 shows data from a unit recorded in the left FB (Figure 2A) during several simulated prey presentations at various angles around the subject's head. Rasters of recordings from this unit are shown from one simulated prey position (Figure 2B). The stimulus moved slowly back and forth at 2 cm/sec. The resulting activity from the unit is lined up according to a zero point as the stimulus passed in front of the mantis's field of view. It should be noted that because the mantis is free to move its head, the angle of the stimulus relative to the mantis's field of view can change. We, therefore, measured the response for all angles and present the raster display for the maximum response angle, in this case 106°. Blue rasters were from trials where the target is moving in a leftward direction, while red rasters were from trials where the target is moving rightward. The histograms below sum the two sets of responses and show a strong response when the stimulus was moving leftward. In contrast, when the stimulus was moving rightward, there was little or no change in activity.

By repeating the procedure reported in Figure 2B at various distances and angles, we were able to produce a contour plot for each unit showing either positive (brown) or negative (blue) correlations associated with movements of the simulated prey (Figure 2C). The positive and negative regions were established by generating STRF plots that relate spike activity with, in this case, target movement (Figure 2D) at various angles and distances around the mantis's head. A positive correlation typically arises from increased spike rate, whereas a negative correlation comes from decreased spike rate. In the STRF plots, spike activity was located at time 0 on the X axis. The plots then showed the correlation between target movement and spike activity. Plots that rise above 2 SDs to the left of the 0 point indicate positions where simulated prey movement preceded an increase in spike activity. Trials where the curve peaked below negative 2 SDs indicate areas where decrease in spike activity followed the prey movement. Any peak that occurred after the 0 point would normally indicate activity that preceded prey movement. For the most part, peaks to the right of 0 were not seen associated with the simulated prey movement. However, in some rare instances (e.g., blue curves in Figure 2Di), a significant peak was carried over to the right of 0. We attribute the carryover in this analysis to the cyclical and continuous nature of the target movement. That is, it may be indicating responses to an earlier target movement with a delay that simply was not captured in the left side peak.

The relationship between the contour plot and the STRF plots is shown by two groups of responses. Activity at various angles around the mantis's head but at the same distance was depicted as 1, 2, 3, or 4 on the contour plot. In Figure 2D, these are indicated as color-coded STRF plots. The dark traces were from specific numbered points on the plot, while the lighter colored curves were from angles that lie on a line between them. In the top set of plots (Figure 2Di), the excitatory region (1) has large peaks (brown curves) to the left of the 0, indicating a strong correlation with the prey movement. In contrast, the blue region (4) in the contour was associated with curves that have large negative peaks to the left of 0, indicating a significant decrease in activity associated with stimulus presentations at these angles. The regions between those extremes (2 and 3) had varying size peaks. Some angles, which encroached on the brown and blue regions of the contour plot during part of the stimulus movement but were centered outside of the main excitatory or inhibitory region, may show weaker responses, while others fail to cross the 2 SD correlation points, indicating no significant response at these angles. The lighter plots showed the transitions between each set of designated points with some reaching significance, while others that were well between the significant regions failed to do so. It should be noted that all of our STRF analysis for this part of the study was conducted on responses that preceded any head movement in order to prevent contamination from proprioceptive or visual responses associated with such actions.

The lower set of plots (Figure 2Dii) shows a similar relationship but now examines the distance from the mantis's head rather than angle. Plots were taken from the line depicted on the contour with points a, b, c, and d indicated. Again, the dark plots were from those designated points, and the lighter ones were at distances between them. Here, the strongest correlations were seen in the b region, which was within the excitatory area of the contour plot (Figure 2C). The positive peaks fell off at distances a and c, which had weaker correlations or failed to reach significance in the STRF plots (Figure 2Dii). At distance d, the STRF plots showed significant negative correlations consistent with the inhibitory region at a greater distance on the contour plot.

**Figure 3:**
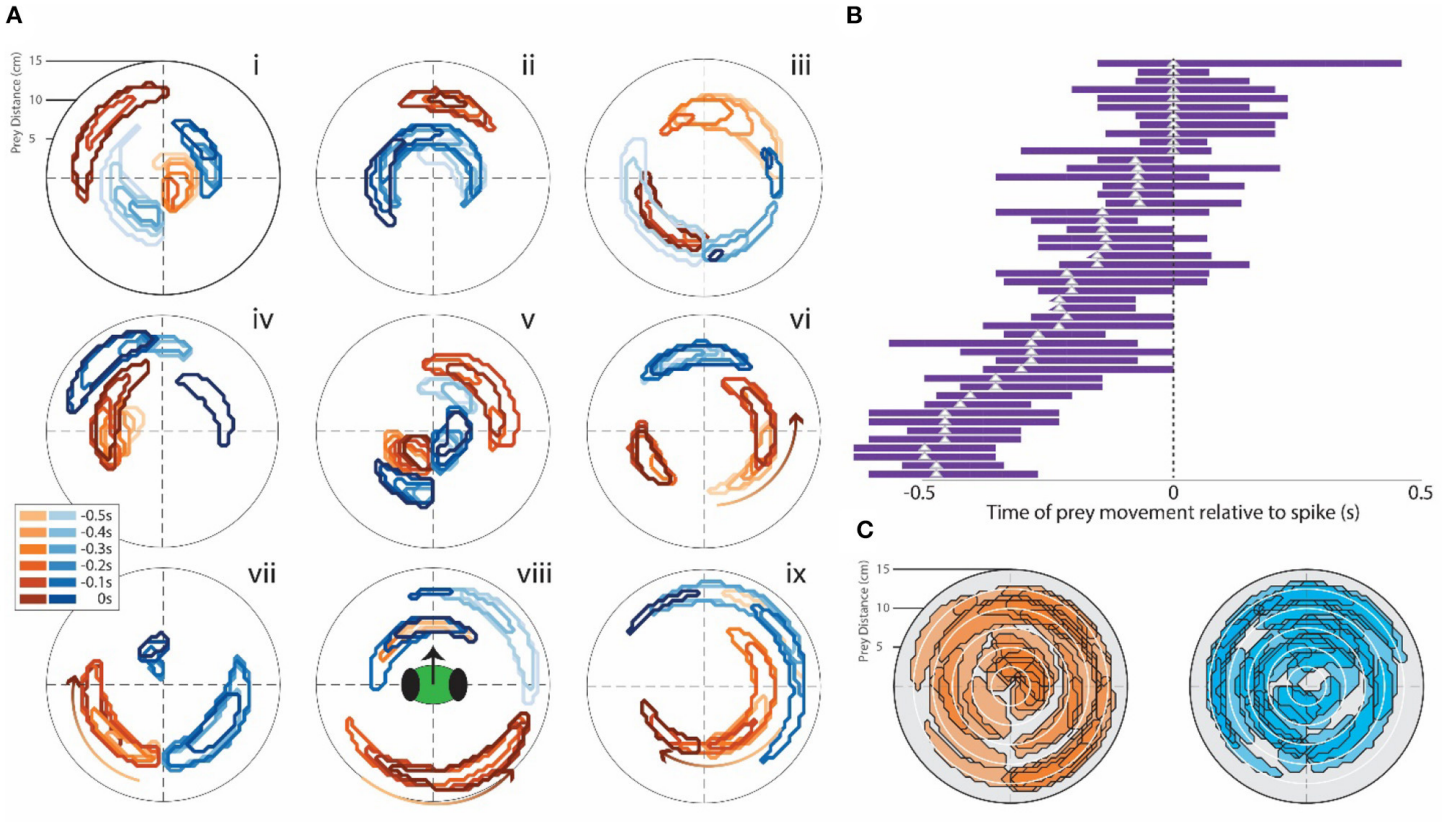
**(A)** Examples of STRF contour plots (as in Figure 2C) for a range of single CX units that responded after the appearance of the target. Note the various different field patterns of positive (brown) and negative (blue) responses to the target. The mantis's head orientation is depicted in contour viii. **(B)** Bars indicate the time period for each neuron where the correlation seen in the STRF curve was above the 2-SD range. The triangle indicates the peak of this curve. All cells that had bars to the left of 0 (point where spike activity changes) responded to target presentation but did not evoke movement of the mantis. These bars represent all cells that showed these response patterns. **(C)** All of the contours we recorded were superimposed on two contour fields: one brown (positive) and one blue (negative). This indicates that the entire area around the head of the mantis is covered by both positive and negative response regions.

Contour plots were generated in this way for all the units recorded in all the 10 experiments. Some had unique properties. Examples are shown in Figure 3. Most are similar to the plots shown in Figure 2B. Many had excitatory (positive weight) or inhibitory (negative weight) regions located at various distances and angles from the mantis. Some units only showed excitation when the simulated prey moved in a particular direction (as was the case in the cell depicted in Figure 2), These were indicated by an arrow on the contours (Figure 3Avi–ix). Forty-eight units recorded in the central complex (specifically all in the FB) showed positive weighting with simulated prey movement in some portion of the visual field. The peak weighting in the generalized linear model of spike generation ranged from 0 to −0.5 s before a spike (Figure 3B), i.e., prey movement precedes spiking in these cells. Contours for other units, including those recorded in the MB, are shown in Supplementary Figure S3. Finally, we considered all CX cells recorded in different animals as a pseudo-population, likely representative of the population in a single animal. When superimposed on one contour plot, the excitatory and inhibitory spatial fields of these cells cover the full range of the visual field tested in these experiments (Figure 3C).

### Responses Associated With Mantis Movement

**Figure 4:**
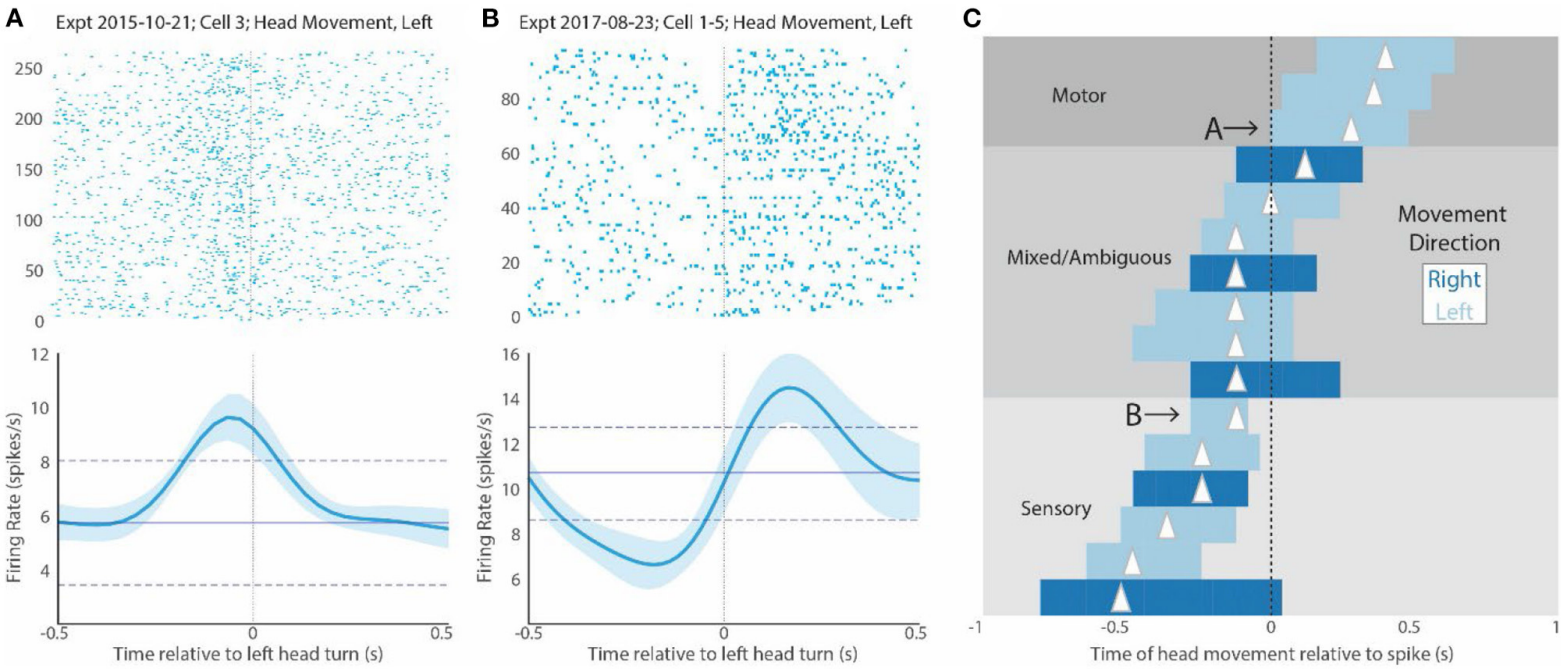
CX activity relative to mantis movement. **(A)** Raster plot of several responses associated with head movement of any kind for a sample cell. The 0 point here indicates the beginning of the head movement. The plot underneath shows the mean and SD firing response of all trials in the raster display. Note that the peak firing precedes the head movement, suggesting that it could contribute to driving the movement. **(B)** Shows a similar display for another cell that responds after the head movement (to the right of 0). This suggests that this cell responds to either proprioceptive or visual inputs associated with the turn or corollary discharge of the motor commands. **(C)** Shows bars indicating the peaks of STRF plots for all cells that were above 2 SDs. As in Figure 3B, the length of the bar indicates duration above 2 SDs, and the triangle indicates the peak of the curve. Dark blue bars are associated with rightward head movements, and light bars are associated with leftward head movements. In this display, bars to the left of 0 indicate regions where head movement precedes increase in spiking (0 point). We have labeled bars that are complete in this region as “sensory.” In contrast, bars to the right of 0 indicate responses where increases in spike activity (0 point) precede movement. We have labeled bars that are complete in this region as “motor”. Bars that span both regions relative to the 0 point are labeled mixed or ambiguous. They could be involved in both motor and sensory effects. Cells whose activity is displayed in **(A,B)** are indicated with letters and arrows. Note that the relationship of the STRF plots relative to the 0 point is the opposite of that seen in the rasters. That is because the 0 point in the STRF plots indicates the point where spike activity increases, whereas the 0 point in the raster display is the point where movement commences.

We next examined the relationship between neural activity and the mantis's own movement (Figure 4). To do this, we digitized the praying mantis's movements in the arena. Most of these were turning movements toward the prey, which occurred in three stages. Target signals near the front of the praying mantis may require only a small rotation of the head to bring the eyes in line with the stimulus. Larger targeting movements require turning of the thorax. Praying mantises have a joint between the prothoracic and mesothoracic segments that allows them to rotate in the horizontal plane. Even larger turns require leg movements that turn the entire body.

A raster display that is now generated relative to the onset of head movement (time 0), shows that CX activity could be associated with these actions. CX units that respond during targeting can fall into two categories. First, they can respond prior to movement (Figure 4A). This effect would be similar to that which was reported in cockroaches, where most of the activity associated with turns or changes in forward stepping preceded movement (Martin et al., 2015). Alternatively, increases in activity could occur after the turn starts (Figure 4B). This type of activity would probably represent some form of reafference. It could stem from visual responses as the head rotates relative to its visual world or from proprioceptive cues monitoring head, thoracic, or leg movements and ascending to the brain. These possibilities are not mutually exclusive. Even where activity preceded movement, the continued activity after the turn commences could result from reafference. It is also possible that the same cell only shows motor control activity for a specific set of targeting movements with reafference occurring elsewhere. Finally, since the target moves back and forth, it is possible that some of the activity during the movement period is associated with motor control associated with reversal of target movement.

To further examine the relationship between CX activity and mantis head movement, we performed STRF analyses on all turning movements made by the mantis during each experiment (Figure 4C). The peaks of each cell can be divided into 3 groups. For cells with STRF plot peaks totally to the right of 0, all significant changes in spike activity preceded the mantis's movements and could initiate such actions. We label these responses as “motor.” For cells with STRF peaks totally to the left of 0, significant activity changes occur during or slightly after movement and probably are associated with reafference or efference copy. Regardless of the source, we refer to these responses as “sensory.” Between these cells are cases where significant STRF peaks span 0. That is, the peak begins to the right but extends to the left of 0. These types of neural responses start before movement and continue to show significant increases during the movement. That is, they could initiate the movement but then receive reafference or efference copy during the movement. We label these responses as “mixed.”

**Figure 5:**
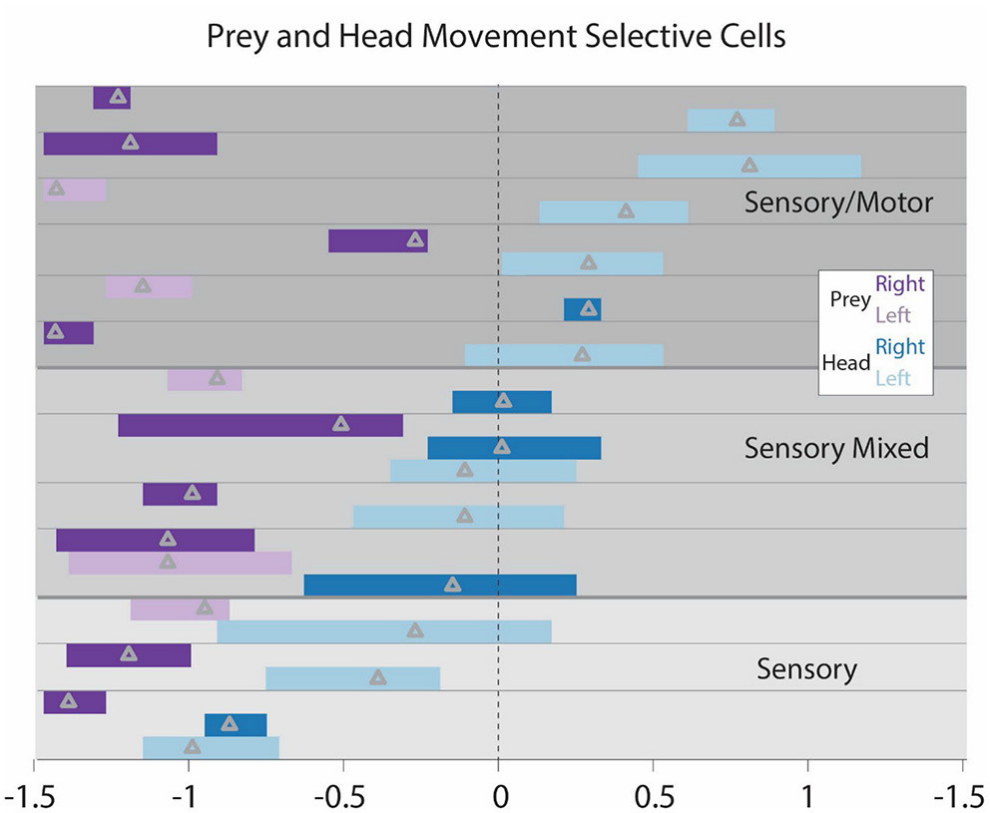
STRF peaks for all cells that both respond to the target movement and respond relative to the praying mantis's movement toward the target. Each cell's peaks are positioned between horizontal lines. Cells are separated into 3 categories. Purely “sensory” cells (bottom 3) have target peaks and mantis head movements to the left. These are cells that respond to the appearance of the target and after the mantis initiates movement. “Sensorimotor” cells (top) have responses associated with target appearance to the left and mantis movement peaks exclusively to the right of 0. These are cells that increase activity with the appearance of the target and have activity significantly increased prior to the movement. “Sensori-mixed” cells (middle) have increased activity associated with target appearance and have increased activity associated with mantis head movement that substantially spans 0. These cells respond to the appearance of the target and have some activity prior to the initiation of mantis head movement that continues throughout the mantis head movement. It should be noted that one sensory and one sensorimotor cells have a slight overlap of the head movement peaks into the opposite region, but we regarded this overlap as minimal. These were subjectively placed in the categories indicated here.

Finally, in turns associated with the simulated targets, we found several cells that both responded to target appearance and were associated with mantis turning movement (Figure 5). These responses fall into 3 categories. Some show responses that attend to the target (magenta bars with peaks to the left of 0) and peaks associated with movement (blue bars) to the left of 0 (re-afference or efference copy). We label these responses “sensory,” since they only seem to include sensory effects relative to the target, whether that be attention to the target appearance or reafference during the movement. Another category has attention peaks (magenta bars) to the left, but mantis movement peaks (blue bars) totally to the right. These would be cells that respond to the target's appearance and then fire before self-movement starts. We label these “sensorimotor.” The remaining cells have peaks associated with target appearance to the left but motor peaks spanning 0 (“mixed” in Figure 4). These cells respond to the target appearance and have activity preceding mantis turning but then continue through the actual turn. The cells in the last two categories could be part of a population of cells that controls the entire predatory stalking behavior. The first group could still contribute to the stalking behavior but only in association with cells in the other groups.

### Movement Evoked by Stimulating Through Tetrodes

**Figure 6:**
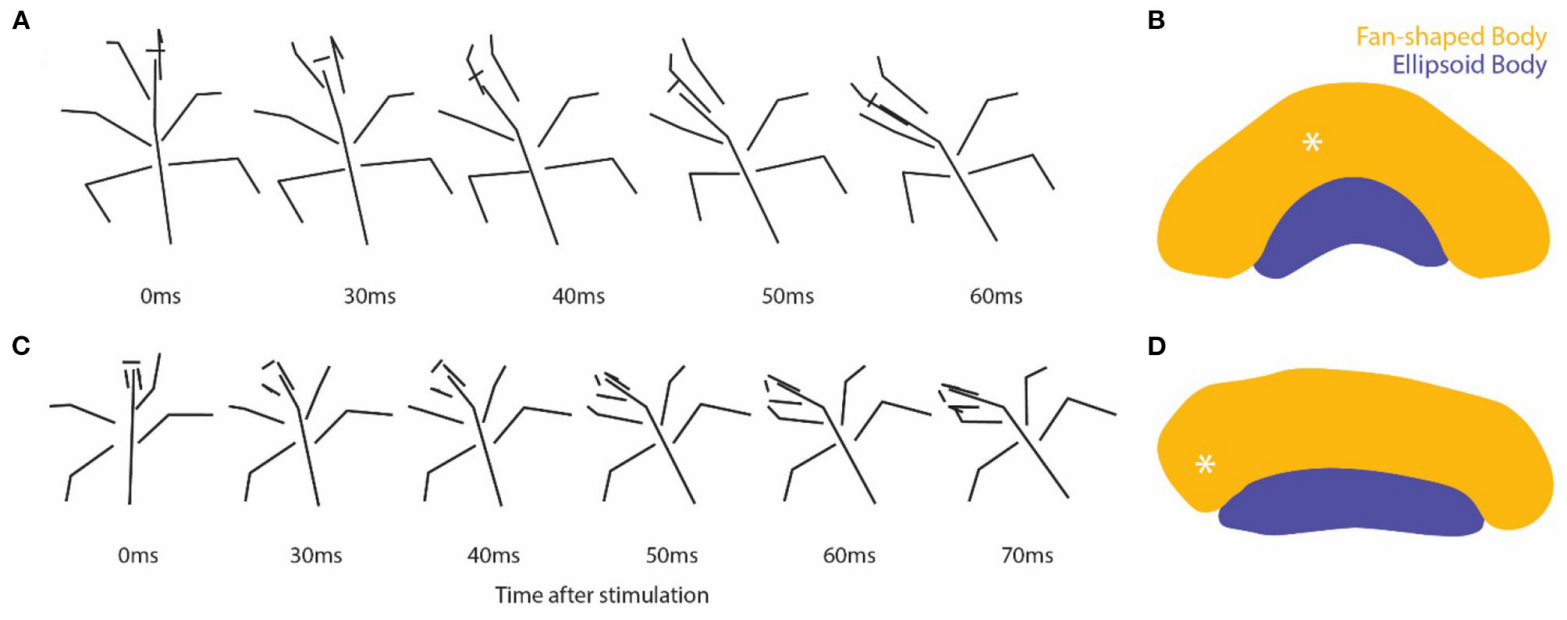
**(A,C)** Stick figures drawn from individual video frames at the time of stimulation through the tetrodes and 4–6 frames 10 ms apart following that point. **(B,D)** Diagrams of sections in the CX showing the location of tetrode tips (asterisk) for each experiment.

We routinely passed currents through the tetrodes at the end of the simulated target experiments. We recorded videos in 5 of the analyzed FB preparations (Figure 6). All 5 evoked turning movements. The stimulation in 4 of them caused movement toward the side on which the tetrodes were located (3 to the left and 1 to the right). The remaining preparation had two tetrodes implanted, one in the left FB and one in the right MB. The stimulation in this preparation caused movement to the right regardless of which tetrode was stimulated. These stimulus-evoked movements verified that the tetrodes were located in regions at least near where neurons that control movement are located (Figure 6). As indicated previously, two-thirds of the tetrodes (8) were shown histologically to be located in the FB. The remaining 4 tetrodes were located in the MB.

## Discussion

The results presented here show that neurons in the CX and, more specifically, in the FB both respond to either real or simulated prey and have the capacity to generate turning movements toward that target. Because we used extracellular recording methods, we cannot say exactly which cells we recorded from in any given preparation. Some could be particularly associated with detecting and following the prey, while others generated the appropriate stalking movements to initiate an attack. Still, others may have played a role in both aspects of the behavior. No cases showed changes in spiking associated with the mantis's actual striking behavior. Rather, the activity we recorded in FB neurons seemed to be associated only with the tracking and stalking of the mantis toward the prey. Once near the prey, the strike appears to be ballistic. While the lack of specific neural identification with this technique is a limitation, there are clear benefits. First, we could record for long periods of time, thereby testing a range of angles and distances to which the mantis responded. Second, the recordings were conducted while the mantis was freely moving. This second point is important. A recent study on monarch butterflies (Beetz et al., 2022) that used tetrode recordings showed that properties of CX neurons changed dramatically as the butterflies transitioned from restrained to active flight as well as to freely rotating flight. Thus, in our study, it was critical to ask questions regarding predatory behavior while the mantis was actually engaged in freely moving behavior.

The details of these relationships may well vary from neuron to neuron in the CX. Since our extracellular recordings do not allow us to identify the source of activity at the cellular level, we may be recording CX cells at numerous levels of motor control. Nevertheless, our analysis shows that at least some CX neurons recorded in the FB respond to both natural and simulated prey, as the praying mantis targets and stalks them. While the cells are likely to have branches in the FB, we cannot be certain that they do not also pass through other CX neuropils. Individual cells are biased to particular regions around the head including angle and distance. At least some are activated prior to targeting movements. This result implies that the CX plays a role in sensorimotor control of this predatory behavior. Whether some or all such CX neurons are restricted to predator attention movements will await future studies. Regardless, this function, in at least some CX neurons, appears to be similar to goal-directed target cells recorded in the hippocampus of bats (Sarel et al., 2017). As with the bat study, the mantis neurons we report on here encode both goal distance and goal direction. However, unlike the bat study, we cannot comment on whether the mantis can locate its target if it is temporarily hidden. It will be interesting to examine to what extent the details of the circuits within a predatory insect's CX that result in attention movements toward prey are similar to the navigational mechanisms that have been proposed for *Drosophila* (Green et al., 2017; Turner-Evans et al., 2017; Hulse et al., 2021) and bees (Stone et al., 2017). Do the circuits used in hunting strategies of predators represent the modified navigational circuits used by other insects in foraging or path integration?

We also made 4 recordings in the MB, and 3 of these provided units that showed significant sensory responses (Supplementary Figure S3). Because of the relatively small number of MB recordings, we did not analyze these units further. Nevertheless, the fact that they responded to the simulated target in manners similar to the CX units suggests that MB neurons may also play some role in targeting prey. The similarities could arise as a result of similar projections from the visual system to the CX and MB neuropils or by communication between these two large brain regions (Mizunami et al., 1998; Hulse et al., 2021).

The lack of precise identification of each studied neuron could result in ambiguities in our results. Since we recorded from these subjects over hours, it is possible that the electrodes moved at some point in the experiment and were actually recording from different neurons. Thus, the motor and sensory roles of a neuron could result from changes in recording site. While our recordings at all four electrodes of a given tetrode appeared to be constant throughout, we cannot completely eliminate that possibility over the course of a long experiment. Nevertheless, in this case, it probably is not a critical issue. Since the mantis was both detecting and turning toward its prey at essentially the same time, it was using both motor and sensory roles almost simultaneously.

### Advantage of Predators for Navigational Studies

Predatory species provide a system in which a clear important target is being sought by a subject. Previous navigational studies focused more broadly on implied navigational systems such as migration of monarch butterflies across North America (Beetz et al., 2022) or moment-to-moment movements of *Drosophila* toward an implied desired direction (Green et al., 2019). However, predators seek a specific target that must be struck in order for them to survive. Not only is the prey target unambiguous, but it can be manipulated by the experimenter either by moving a prey item or, as in our study, mimicking it with a simulated target.

Moreover, that target may be dynamic in itself. The prey will not necessarily remain at a given point but may move through its environment either before being detected or in response to the predator's movements. Problems facing relatively small animals, such as insects as they track moving targets against a background, have been reviewed elsewhere (Gonzalez-Bellido et al., 2016). Two very different strategies have been noted. One is a relatively simple or classical pursuit in which the predator aligns its stalking movements directly toward the prey. This appears to be the strategy that the praying mantises followed in our study. The mantis detects a specific prey item, turns toward it, and closes the distance in order to get near enough the prey for a successful strike. If the prey moves, the mantis simply follows it. In contrast, another well-studied predatory insect, the dragonfly, actually uses an internal model to predict the movements of its prey (Mischiati et al., 2015). In the dragonfly, predictive properties have even been demonstrated at the level of central brain neurons called small target motion detectors (STMDs) recorded intracellularly in restrained insects (Wiederman et al., 2017). Such a system is particularly important for a flying insect making rapid navigational adjustments toward flying prey. It does not appear that such predictions are being made in the slower stalking movements of the praying mantis; however, some predictions could also be made here. Future studies should test this notion.

Our live predator experiments revealed another property that is specific to predator-prey interactions. In order to be successful, the predator cannot constantly switch its attention between targets. Thus, as seen in our Supplementary Video S2, once the mantis focuses on one prey item (the one attempting to right itself in the middle of the arena), both the mantis's movements and the CX neuron that we were recording appeared to ignore another cockroach that walked through the mantis's field of view. Since this second cockroach was tracked earlier in the video, it clearly met the criteria for attack.

The selective attention we saw in that video could be explained by “competitive selection,” as described in intracellular recordings of STMD neurons of the dragonfly central brain (Wiederman and O'Carroll, 2013). In that study, neural activity was recorded in response to two very different target presentations. Then, both target patterns were presented simultaneously. Rather than producing a response characteristic of the combined patterns for the two targets, the neuron's response locked on to one or the other pattern. In some cases, the pattern would switch from one to the other during the trial, suggesting a competition between patterns that could change during presentation. Indeed, a more recent study used frequency-tagged intracellular trains to identify which target of a pair is selected at any moment (Lancer et al., 2019). It will be interesting to see in both restrained and freely moving preparations what target properties result in both consistency and switching, an issue that is critically important in many animals, particularly in hunting behaviors.

### Why Go Through a Region Like the CX?

The latter factors point to issues regarding the role of the CX in the navigational movements that we examined here and those that others have studied elsewhere. One can ask why the insect brain requires something as complex and highly organized as the CX to make directed movements. If an insect was only required to move toward a target, it could accomplish this with a simpler system such as the flip-flop interneurons that were described earlier in dragonflies (Olberg, 1981). However, navigational movements are often affected by many other context-dependent factors. For example, a predator such as the praying mantis may alter its hunting strategy depending on its level of hunger. A recent study demonstrated that higher satiety reduced the distance and angles around the mantis's head to which it would respond (Bertsch et al., 2019). That is, starved mantises stalked much more distant prey. However, as they fed, the distance that they responded to decreased with each meal. Eventually, the mantis switched to an ambush strategy, in which it would strike a prey item that came near but would not actively move toward it.

The effect of satiety on hunting strategy could also be generated in starved mantises by injecting insulin into its abdomen. Insulin is a hormone that increases in response to feeding in a wide range of animals (Mattila and Hietakangas, 2017). Thus, it is likely that neurons in the CX are altered by such hormonal effects. Indeed, a wide range of neuromodulators are known to be present and have effects within the CX (Homberg, 2002; Nässel and Homberg, 2006; Kahsai and Winther, 2011), and are known to alter behavior (Kahsai et al., 2010).

Other factors that can affect directional movement include proprioceptive inputs such as those from the haltere balance organs of flies. The activity from these structures is now known to influence CX neurons (Kathman and Fox, 2019). Moreover, the change in compass properties seen in the monarch butterfly as it transitions from restrained conditions to rigid and then flapping flight appear to stem, at least in part, from efference copy that, again, seems to alter CX activity (Beetz et al., 2022).

The example of context-dependent effects on directional behavior points to possible reasons for movement control to pass through a complex structure like the CX. It also points to the importance of recording techniques that allow one to monitor freely moving behavior over long periods of time even as conditions change. The tetrode recording technique used in this study and the STRF analysis that allowed us to relate CX unit activity with movement of either the prey target or the mantis itself toward that target provide such tools.

In the long run, neuroethological studies seek to explain the neural basis of complex behaviors. As has been pointed out previously, neuroethology in itself brings together two complimentary but often hostile disciplines (Ritzmann and Fox, 2022). Ethologists have traditionally sought to describe behavior while restricting any influence that the observer might impart on the subjects of interest. At the opposite extreme, neurobiologists often try to record in isolated neural structures or highly restrained preparations in order to describe very precise neural properties in identified neurons. Neuroethologists typically attempt to address this conflict by working through stepping stones from descriptions of behavior to ever more restrained preparations. They may arrive at restrained intracellular recordings but only after taking smaller technical steps between the freely moving behavior and a restrained preparation. The tetrode recordings used here and elsewhere provide an important stepping stone between these extremes. In practice, they would serve to link behavioral data to restrained experiments with identified neurons or vice versa.

## Data Availability Statement

The raw data supporting the conclusions of this article will be made available by the authors, without undue reservation.

## Author Contributions

AW, JM, RR, and GS: conception of study. RR, GS, JM, and AW: funding. JM: programs for experimental control and analysis of data. JM and AP: recording live prey experiments. AW: recording simulated prey experiments and processing data. AP: histology. RR and JM: writing manuscript. RR, JM, AW, AP, and GS: editing manuscript. All authors contributed to the article and approved the submitted version.

## Funding

This material is based on a study supported by the National Science Foundation under (Nos. IOS-1557228 to RR, IOS-1557279 to GS, and IIS-1704366 to JM) and by the German Research Foundation under Fellowship (No. Wo2000/1-1 to AW).

## Conflict of Interest

The authors declare that the research was conducted in the absence of any commercial or financial relationships that could be construed as a potential conflict of interest.

## Publisher's Note

All claims expressed in this article are solely those of the authors and do not necessarily represent those of their affiliated organizations, or those of the publisher, the editors and the reviewers. Any product that may be evaluated in this article, or claim that may be made by its manufacturer, is not guaranteed or endorsed by the publisher.

## Supplementary Material

Supplementary Video S1 Sample video of mantis responding to simulated targets. Time stamps of a single unit are depicted at the bottom and as audio.

Supplementary Video S2 Video taken of a freely moving mantis with tetrode implants hunting cockroach nymphs. Time stamps of one unit are depicted running at the bottom and as audio.

Supplementary Figure S1 Diagram showing the location of all tetrode recording sites in the fan-shaped body (FB) and mushroom body (MB). White dots are from single tetrode experiments and gray from dual tetrode experiments. Each of the light and dark gray pairs is from the same experiment. The various neuropils are labeled with colors.

Supplementary Figure S2 **(A)** Procedure for spiking model estimation. The position of the prey stimulus and head angular velocity (1) are used to generate a spatiotemporal response function for several values of delay before and after a spike in the recorded neuron (2). Linear spatial temporal receptive fields (STRFs) are combined with a nonlinear spike-generating function (3), and the resulting modeled spike train is compared with the actual recorded spike train (4). The STRFs are iteratively modified to reduce the difference between modeled and real spike trains (5). **(B)** Time series is generated for a single spatial bin (white circles) in the (STRF) at various times relative to the spike occurrence at time 0. The normalized value of the weight for the spatial bin at each spike delay is plotted in the graph on the right. The weight is analogous to the correlation between a simulated prey stimulus at that location and the neuron producing a spike at some time before or after the prey is in that location. Larger, positive values for this weight at negative delays (left side of the graph) mean that the prey in this location was associated with the neuron producing increased numbers of spikes after the prey moved. Larger, positive values at positive delays (right side of the graph) mean that the associated event was likely to occur after the neurons produced a spike. This would be seen, i.e., when a motor neuron is involved in generating a movement (refer to Figure 4 in the main text). Negative peaks at either of these time frames would suggest a decrease in spike activity associated with movement.

Supplementary Figure S3 Additional cells with significant responses to prey location. **(A)** Contour maps from cells recorded in the central complex (CX) beyond those depicted in Figure 3A. **(B)** Contour maps from cells recorded in the mushroom body (MB). In both cases, the borders group the maps from cells recorded from the same tetrode in the same preparation.
