# Supplementary figures and images for: The Role of Central Complex Neurons in Prey Detection and Tracking in the Freely Moving Praying Mantis (Tenodera sinensis)
Source: Front Neural Circuits. 2022 Jun 13;16:893004. doi: 10.3389/fncir.2022.893004 (PMC9234402; doi:10.3389/fncir.2022.893004)

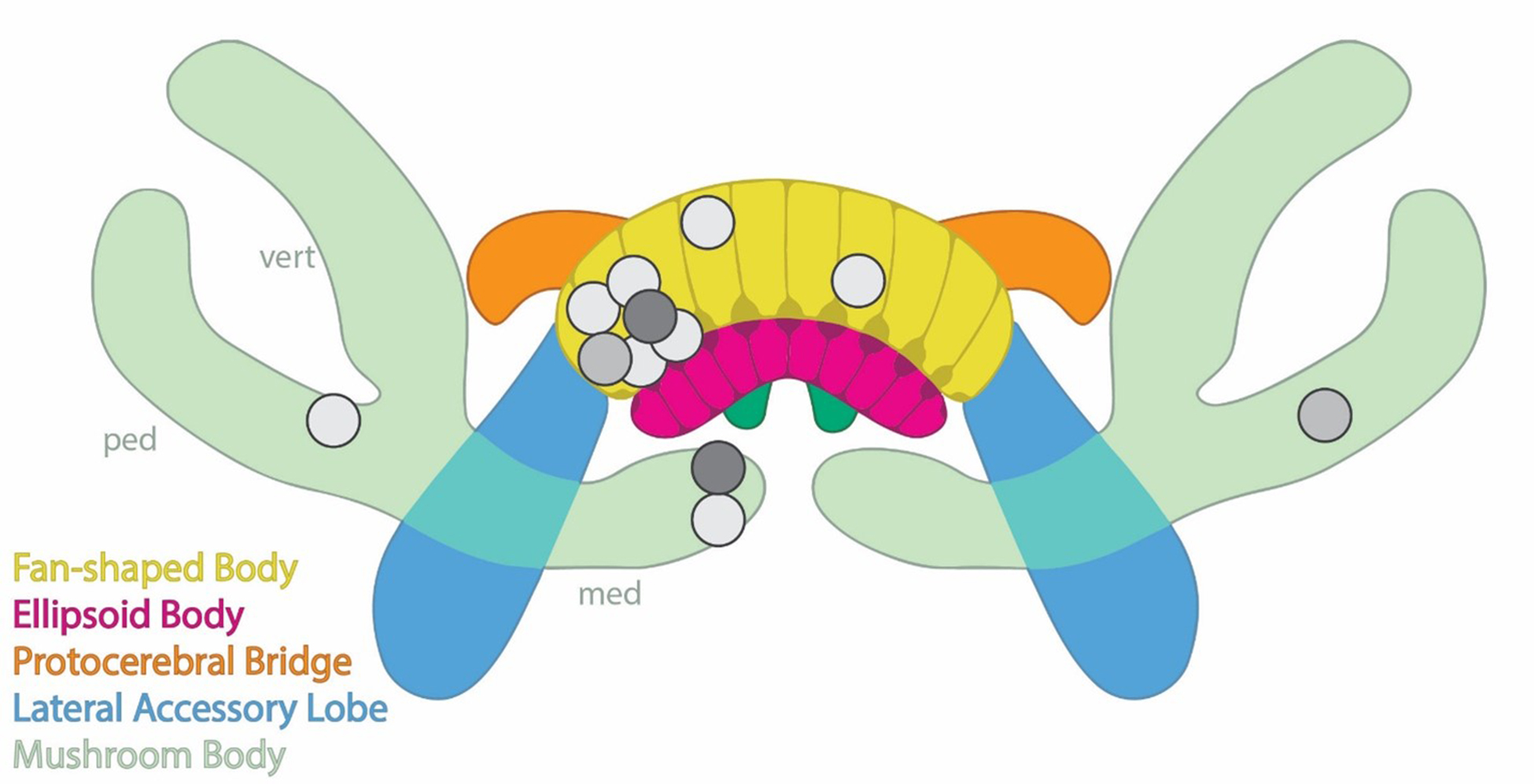

Supplement: Supplementary Figure S1 — Diagram showing the location of all tetrode recording sites in the fan-shaped body (FB) and mushroom body (MB). White dots are from single tetrode experiments and gray from dual tetrode experiments. Each of the light and dark gray pairs is from the same experiment. The various neuropils are labeled with colors. [file Image_1.JPG]

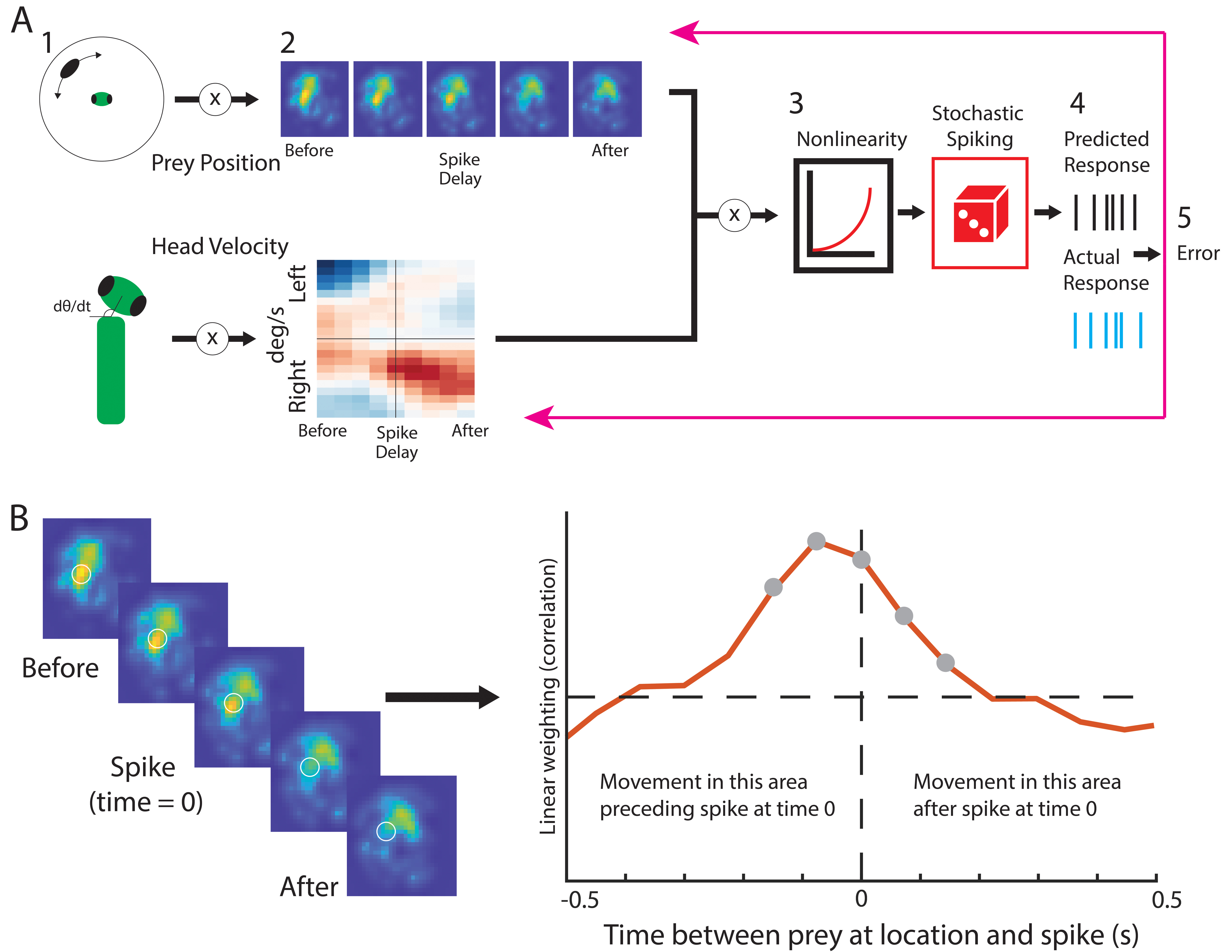

Supplement: Supplementary Figure S2 — (A) Procedure for spiking model estimation. The position of the prey stimulus and head angular velocity (1) are used to generate a spatiotemporal response function for several values of delay before and after a spike in the recorded neuron (2). Linear spatial temporal receptive fields (STRFs) are combined with a nonlinear spike-generating function (3), and the resulting modeled spike train is compared with the actual recorded spike train (4). The STRFs are iteratively modified to reduce the difference between modeled and real spike trains (5). (B) Time series is generated for a single spatial bin (white circles) in the (STRF) at various times relative to the spike occurrence at time 0. The normalized value of the weight for the spatial bin at each spike delay is plotted in the graph on the right. The weight is analogous to the correlation between a simulated prey stimulus at that location and the neuron producing a spike at some time before or after the prey is in that location. Larger, positive values for this weight at negative delays (left side of the graph) mean that the prey in this location was associated with the neuron producing increased numbers of spikes after the prey moved. Larger, positive values at positive delays (right side of the graph) mean that the associated event was likely to occur after the neurons produced a spike. This would be seen, i.e., when a motor neuron is involved in generating a movement (refer to Figure 4 in the main text). Negative peaks at either of these time frames would suggest a decrease in spike activity associated with movement. [file Image_2.JPG]

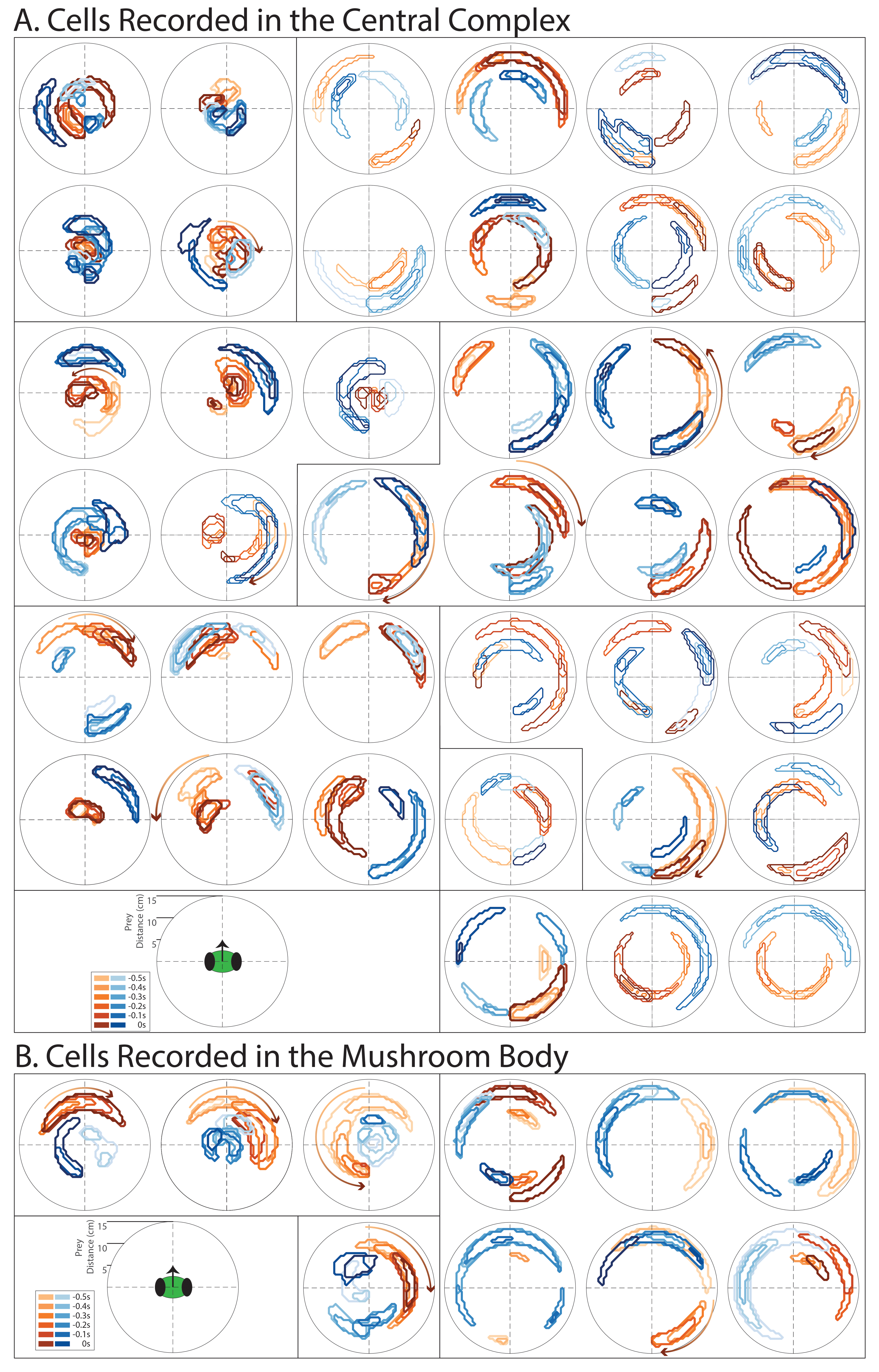

Supplement: Supplementary Figure S3 — Additional cells with significant responses to prey location. (A) Contour maps from cells recorded in the central complex (CX) beyond those depicted in Figure 3A. (B) Contour maps from cells recorded in the mushroom body (MB). In both cases, the borders group the maps from cells recorded from the same tetrode in the same preparation. [file Image_3.JPG]
